# Long-term unmet supportive care needs of teenage and young adult (TYA) childhood brain tumour survivors and their caregivers: a cross-sectional survey

**DOI:** 10.1007/s00520-021-06618-7

**Published:** 2021-10-12

**Authors:** Emma Nicklin, Galina Velikova, Adam Glaser, Michelle Kwok-Williams, Miguel Debono, Naseem Sarwar, Florien Boele

**Affiliations:** 1 Leeds Institute of Medical Research at St James’s, University of Leeds, Leeds, UK; 2 Patient-Centred Outcomes Research Group, St James’s Institute of Oncology, Level 6, Bexley Wing, Beckett Street, Leeds, LS9 7TF UK; 3 Leeds Teaching Hospitals NHS Trust, Leeds, UK; 4 Sheffield Teaching Hospitals NHS Foundation Trust, Sheffield, UK; 5 University of Leeds, Leeds Institute of Health Sciences, Leeds, UK

**Keywords:** Brain tumour, TYA, Family caregiver, Supportive care, Unmet needs, Quality of life

## Abstract

**Introduction:**

The supportive care needs of long-term childhood brain tumour survivors, now teenagers and young adults (TYAs), and their caregivers are largely unknown. We aimed to describe their supportive care needs and explore associations between needs and quality of life (QoL).

**Methods:**

Participants were recruited from long-term follow-up clinics (in three NHS Trusts in England) and online. Participants included childhood brain tumour survivors, ≥ 5 years from diagnosis, currently aged 13–30, and their primary caregivers. Survivors completed the Supportive Care Needs Survey (SCNS) Short Form and caregivers the SCNS-Partners & Caregivers, alongside validated QoL questionnaires (Peds-FACT-Br and CQOLC).

**Results:**

In total, 112 individuals (69 survivors/43 caregivers) participated. Survivors reported on average 9.4 (± 8.5) unmet needs. Needs were greatest in the psychological domain, with anxiety (60.3%), uncertainty about the future (50.7%) and feeling down and depressed (48.5%) most commonly reported. Caregivers reported on average 12.4 (± 12.3) unmet needs. Again, the greatest number of unmet needs was observed in the psychological domain. Many caregivers also reported information needs around financial support/government benefits (42.9%) and possible survivor fertility problems (42.9%). Multivariable linear regression analysis showed that female survivors, unemployed survivors, survivors further away from diagnosis and single caregivers were more likely to report unmet needs. More unmet needs were significantly associated with poorer QoL in survivors and caregivers.

**Conclusion:**

This research provides leads to improving supportive care and long-term follow-up services. Psychological support represents the biggest gap in care. Understanding unmet needs and recognising what services are required are critical to improving quality of long-term survival.

**Supplementary Information:**

The online version contains supplementary material available at 10.1007/s00520-021-06618-7.

## Introduction

Childhood brain tumours are the second most common cancer after leukaemia and the most common solid tumour [1]. Advances in treatment have meant that the survival of children treated for brain tumours has improved significantly [2, 3]. Recent statistics report that the 5-year survival rate for all childhood brain tumours is 73.3% and 10-year survival is 69.9% [2].

Improvements in survival rates are encouraging, yet the quality of extended survival is equally important. Teenage and young adult (TYA) survivors of childhood brain tumours are an ever-growing population, many of whom live with late effects as a consequence of their tumour and treatment [4, 5]. Young adulthood is a period of change, dramatically characterised by sexual maturity, hormonal activity, rapid physiological development and complex emotional change. For most young people, these developmental years are profound and important. However, TYA childhood brain tumour survivors are often faced with tensions between their emerging abilities, and their reduced capabilities imposed by their tumour and treatments [6].

Responsibility for caring and supporting survivors is often met by their immediate family, usually parents. This caring role often continues into long-term survivorship and is complicated by survivors unique late effects, including neurocognitive deficits, physical disabilities and social issues [6]. Previous research has suggested that caregivers of brain tumour survivors may have worse quality of life than caregivers of other cancer groups [7], and experience greater stress and worse parental mental health than parents with children without health problems [8, 9]. Previous studies in other cancer groups have also found associations between aspects of caregiver wellbeing and patient survival [10], outcomes and well-being [11, 12]. Supporting caregivers to perform their responsibilities, while keeping their own physical and emotional health, is essential.

Supportive care needs in cancer survivors are diverse and fall into numerous domains, such as physical, psychological, practical and information. Here we define ‘needs’ as having ‘*the requirement of some action or resource that is necessary, desirable or useful to attain optimal well-being*’ (Foot, 1996, as cited in Sanson-Fisher, et al., 2000, p.227) [13].

Currently, there is insufficient knowledge of what TYA brain tumour survivors and their caregivers specifically need from supportive care [6]. To better services, having a clear overview of unmet supportive care needs this population is crucial — yet, we found little research addressing this in our recent systematic review [14]. TYA brain tumour survivors are a unique patient cohort with specific challenges and vulnerabilities, including the desire to gain independence, educational issues and exploring romantic relationships [14]. It is important that their needs are accessed separately from children or older adult survivors as their needs will be influenced by unique developmental issues, life milestones and other age-related issues.

Therefore, we aimed to (1) describe the unmet supportive care needs of TYA survivors of childhood brain tumours and their caregivers, (2) explore if sociodemographic/clinical data are associated with unmet needs and (3) determine whether unmet needs are associated with quality of life (QoL) outcomes.

## Methods

### Study design

These findings are from the quantitative phase of a mixed-methods study. The qualitative phase is described elsewhere [14]. In this paper, we report the quantitative phase which consisted of a cross-sectional survey. We used the Strengthening the Reporting of Observational Studies in Epidemiology (STROBE) checklist when writing the report [15].

Throughout the study a Patient Advisory Group was consulted to ensure that the research remained patient-centred and appropriate. Members were sought through a national brain tumour charity — *brainstrust*. The group consisted of three parent caregivers and one childhood brain tumour survivor aged 15 years old. The representatives provided feedback (in face-to-face meetings and via email) on study aims, study methods (e.g. the best ways to collect data, outcome measures), study materials (e.g. checking the clarity of language used in information sheets) and study outcomes (e.g. types of dissemination).

### Study population and recruitment

Participants were eligible if they were primary brain tumour survivors: currently aged 13–30, diagnosed before 14 years of age and at least 5 years from diagnosis. Globally the age range for TYAs is highly variable [16, 17]. The lower age limit was chosen as in the UK, the lower limit for TYA supportive care is generally defined as 13 years of age [17, 18]. In the UK, the upper limit is normally defined as 24 years of age [17, 18]. However, there is an argument that this is too low, as the transition to adulthood is becoming prolonged [19]. For example, the age of first marriage is higher than in previous decades. Therefore, as guided by our Patient Advisory Group we set the upper age boundary to 30. Caregivers were eligible if identified as the primary caregiver of the survivor. Survivors were not eligible if they suffered serious cognitive dysfunction impeding their ability to complete study procedures.

Recruitment took place from October 2018 to February 2020. Participants were recruited from three long-term follow-up National Health Service (NHS) clinics in Yorkshire, England. Survivors and caregivers who gave informed consent were asked to complete a self‐report survey about their own experiences either online or on paper. Support with survey completion was offered if needed (e.g. some survivors required the survey to be read to them due to poor eye sight). Survivors could take part without caregivers and vice versa.

In addition, an online version of the survey was advertised with help from three brain tumour charities (The Brain Tumour charity, *brainstrust* and Yorkshire’s Brain Tumour Charity).

### Measures

#### Sociodemographic and clinical characteristics

Survivor and caregiver sociodemographic characteristics were obtained through self‐report questionnaires. The survivor’s clinical characteristics (e.g. brain tumour diagnosis and treatment) were obtained through medical record review. Participants who were recruited online were asked to self-report this data.

#### Supportive care needs

Survivor needs were measured using the Supportive Care Needs Survey Short Form (SCNS-SF34) [20]. The 34-item instrument assesses needs across five domains: psychological, physical and daily living, health system and information, patient care and support, and sexuality. The instrument previously demonstrated excellent internal consistency (α = 0.86 to 0.96), reliability (α = 0.90 to 0.95) and acceptable convergent validity (*r* = 0.48–0.56) in adult cancer patients. Caregiver needs were measured using the Supportive Care Needs Survey for Partners & Caregivers (SCNS-P&C) [21]. The SCNS-P&C is a 45-item instrument. Items are grouped into four domains: health-care service needs, psychological and emotional needs, work and social needs and information needs. This scale has demonstrated high internal consistency (α = 0.70) and reliability (α = 0.88–0.94) in caregivers of cancer patients [21].

Both SCNS questionnaires are answered by a 5‐point Likert scale (1 = no need/not applicable; 2 = no need; satisfied; 3 = low need; 4 = moderate need and 5 = high need). A standardised Likert scale score was calculated for each domain, with a possible 0 to 100 range. High scores indicated higher unmet needs [22].

#### Quality of life

Survivor’s QoL was measured using the Paediatric Functional Assessment of Cancer Therapy — Brain (Peds-FACT-Br), the adolescence specific module [23]. The Peds-FACT-Br (Adolescence) was chosen because it was disease specific (for brain tumour survivors) while also being age specific (for TYAs), and because the Patient Advisory Group preferred it over other tools. The Peds-FACT-Br has adequate internal consistency (α ≥ 0.75) and reliability (α = 0.69) when tested in childhood brain tumour survivors [24]. Caregivers’ quality of life was measured using The Caregiver Quality of Life Index–Cancer (CQOLC) [25]. The CQOLC possesses good internal consistency (α = 0.91), test–retest reliability (α = 0.95) and acceptable convergent validity (*r* = 0.08–0.64) when tested on cancer caregivers [25]. Scores could range from 0 to 148 (Peds-FACT-Br) and 0 to 140 (CQOLC), a higher score indicates better quality of life.

### Data analysis

All analyses were performed using SPSS V.23. Descriptive analysis was used for sociodemographic and clinical characteristics as well as supportive care needs items. In addition, the mean summated scores from each domain in the SCNS-SF34 and SCNS-P&C were calculated to understand which domains scored the highest in relation to participants requiring the most help [22].

Univariable linear regression analyses were performed to explore associations between total unmet needs/individual domain scores (dependent variable) and independent variables: sociodemographic (e.g. age, sex and employment status) and survivor clinical characteristics (e.g. time since diagnosis and treatment). Variables selected were informed by our systematic review [6], but as little prior research exists, it was also in part explorative and guided by the limits of the data, e.g. there were too many categories for brain tumour diagnosis and location. Variables associated at *p* = 0.10 level were entered into a backward multivariable linear regression analysis.

The Pearson’s correlation coefficient was used to assess the relationship between total number of unmet needs and total QoL score. Values between ± 0 and 0.3 indicate a weak relationship, values between ± 0.3 and 0.7 indicate a moderate relationship and values between ± 0.7 and 1.0 indicate a strong relationship [26]. The overall QoL score was also correlated against the SCNS-SF34 and SCNS-P&C domains. A two-sided *p* value of 0.05 was considered statistically significant.

Missing data were less than 5%. Missing data were not replaced in the descriptive analyses. In the regression and correlation analyses where questionnaire subscale/domain scores were needed, the scores were prorated when less than half of the items within a domain were missing [27]. Where there were more missing data, the participant’s data for that scale or total score were excluded from the analysis.

## Results

### Sociodemographic and clinical characteristics of participants

In total, 112 participants completed the survey (69 survivors and 43 caregivers). Seventy-eight survivors and 53 caregivers were identified in long-term follow-up clinics and invited to partake in the survey, 50 survivors and 32 caregivers completed the survey (response rate = 64.1%/60.4%). Only one survivor with severe cognitive deficits (which clinical staff believed would prevent successful completion of study procedures) was not approached. A further 19 survivors and 11 caregivers were recruited online through charities.

Table 1 provides a summary of participants’ sociodemographic and clinical characteristics. There were more male survivors (53.6%), 40.5% were in some form of employment, over a third were unemployed/unable to work (33.3%) and the majority were single (79.7%). The mean age at diagnosis was 7.2 years and on average it was 17.4 years since their diagnosis. Diagnoses were varied; medulloblastomas (34.8%) and astrocytomas (26.1%) were the most common.

**Table 1:** Survivor and caregiver sociodemographic and clinical characteristics

	Survivors	Caregivers
Sex *N* (%)		
Male	37 (53.6)	6 (14.0)
Female	32 (46.4)	37 (86.0)
Ethnicity *N* (%)		
White British	68 (98.6)	40 (93.0)
Other	1 (1.4)	1 (2.3)
Missing	0 (0)	2 (4.7)
Age M ( ±), range	22.6 (4.3), 13–30	52.4 (6.4), 37–64
Highest education achievement *N* (%)		
High school	14 (20.3)	12 (27.9)
College	25 (36.2)	7 (16.3)
University	27 (39.1)	15 (34.9)
Masters	0 (0)	1 (2.3)
Other	3 (4.3)	8 (18.7)
Employment *N* (%)		
Working full-time	15 (21.7)	10 (23.3)
Working part-time	13 (18.8)	14 (32.6)
Unable to work due to illness/disability	12 (17.4)	1 (2.3)
Caring for home/family	0 (0)	13 (30.2)
Unemployed	11 (15.9)	1 (2.3)
Student	15 (21.7)	0 (0)
Other	1 (1.4)	4 (9.3)
Missing	2 (2.9)	1 (2.3)
Relationship status *N* (%)		
Single	55 (79.7)	9 (20.9)
In a relationship	14 (20.3)	34 (79.1)
Survivor age at diagnosis *N* (%)		
0–4	22 (31.9)	14 (32.6)
5–10	31 (44.9)	23 (53.5)
11–14	16 (23.2)	6 (14.0)
Time since diagnosis in years M ( ±), range	17.4 (4.9), 7–27	14.1 (5.0), 5–27
Tumour type *N* (%)		
Medulloblastoma	24 (34.8)	16 (37.2)
Astrocytoma	18 (26.1)	11 (25.6)
Craniopharyngioma	6 (8.7)	1 (2.3)
Pineal tumour	4 (5.8)	1 (2.3)
Choroid plexus carcinoma	4 (5.8)	2 (4.7)
Ependymoma	3 (4.3)	3 (7.0)
Other	10 (14.5)	9 (20.9)
Tumour location *N* (%)		
Posterior fossa	15 (21.7)	9 (20.9)
Cerebellum	9 (13.0)	6 (14.0)
Pineal	7 (10.1)	4 (9.3)
Brain stem	7 (10.1)	1 (2.3)
Optic nerve	4 (5.8)	6 (14.0)
Cerebrum	4 (5.8)	3 (7.0)
Brain not otherwise specified	4 (5.8)	3 (7.0)
Other	13 (18.8)	9 (14.0)
Missing	6 (8.7)	3 (7.0)
Tumour grade (at diagnosis) *N* (%)		
Grade I	20 (29.0)	10 (23.3)
Grade II	6 (8.7)	3 (7.0)
Grade III	4 (5.8)	5 (11.6)
Grade IV	18 (26.1)	13 (30.2)
Unknown	21 (30.4)	12 (27.9)
Treatment (ever) *N* (%)		
Resection	51 (73.9)	32 (74.4)
Re-resection	7 (10.1)	4 (9.3)
Radiotherapy	47 (68.1)	31 (72.1)
Chemotherapy	42 (60.9)	34 (79.1)
Posterior fossa syndrome *N* (%)		
Yes	4 (5.8)	3 (7.0)
No	62 (89.9)	40 (93.0)
Not sure	2 (2.9)	0 (0)
Missing	1 (1.4)	0 (0)
Quality of life score M ( ±), range	93.8 (28.1), 33–139	63.19 (27.6), 14–117

Caregivers were mainly mothers (86.0%), a third (34.9%) were educated to university degree level and around half (55.9%) were in full or part-time employment. The majority were married (74.4%); around a fifth (20.9%) were not in a relationship.

### Supportive care needs

Survivors on average reported 9.4 (range 0–30) unmet needs. Caregivers reported more unmet needs on average 12.4 (range 0–42). Table 2 details the percentage of survivors and caregivers experiencing at least one, three, five, ten or fifteen unmet needs. Overall, over three-quarters of survivors (78.3%) reported at least three unmet needs. And over a quarter of survivors (27.5%) had at least fifteen unmet needs. Fifteen (21.7%) reported no unmet supportive care needs.

**Table 2:** Frequency of survivor and caregiver unmet needs

Survivor unmet needs (answering 3–5 on each item)	Survivor *N* = 69 (%)	Caregiver *N* = 41 (%)
No unmet needs	15 (21.7)	5 (11.6)
At least one unmet need	54 (78.3)	38 (88.4)
At least three unmet needs	53 (76.8)	31 (72.1)
At least five unmet needs	45 (65.2)	30 (69.8)
At least ten unmet needs	28 (40.6)	20 (46.5)
At least fifteen unmet needs	19 (27.5)	15 (34.9)

Tables 3 and 4 present the percentages of unmet supportive care needs by domain, as assessed with the SCNS questionnaires in survivors and caregivers, respectively. Psychological unmet needs were most prominent for survivors, with the highest mean domain score (30.2 ± 23.9) as well as the majority of the top 10 ranked unmet needs (7/10). The top three unmet needs were all in the psychological domain: ‘*Anxiety*’ (60.3%); ‘*Uncertainty about the future*’ (50.7%) and ‘*Feeling down or depressed*’ (48.5%). The standardised scores were lowest in the sexuality domain (13.4 ± 19.5). The items with the lowest level of unmet needs were also in the sexuality domain: ‘C*hanges in sexual relationships*’ (10.4%) and ‘*Changes in sexual feelings*’ (13.4%).

**Table 3:** Survivor unmet supportive care needs by domains and individual items of the SCNS-SF34

SCNS-SF34 items by domain	Standardised mean ( ±)*	Survivor with unmet need/number of respondents (%)	Top 10 needs
**Psychological**	30.2 (23.9)		
Anxiety		41/68 (60.3)	1/10
Feeling down or depressed		33/68 (48.5)	3/10
Feelings of sadness		32/68 (47.1)	= 4/10
Fears about the cancer spreading		11/68 (16.2)	
Worry that the results of treatment are beyond your control		15/68 (22.1)	
Uncertainty about the future		34/68 (50.7)	2/10
Learning to feel in control of your situation		26/67 (38.8)	7/10
Keeping a positive outlook		25/67 (37.3)	8/10
Feelings about death and dying		12/67 (17.9)	
Concerns about the worries of those close to you		28/67 (41.8)	6/10
**Physical and daily living**	28.0 (20.0)		
Pain		16/68 (23.5)	
Lack of energy/tiredness		32/68 (47.1)	= 4/10
Feeling unwell a lot of the time		22/68 (32.4)	
Work around the home		14/68 (20.6)	
Not being able to do the things you used to do		23/68 (33.8)	
**Patient care and support**	25.1 (18.2)		
More choice about which cancer specialist you see		9/67 (13.4)	
More choice about which hospital you attend		11/66 (16.7)	
Reassurance by medical staff that the way you feel is normal		18/67 (26.9)	
Hospital staff to attend promptly to your physical needs		13/67 (19.4)	
Hospital staff to acknowledge, and show sensitivity to, your feelings and emotional needs		12/67 (17.9)	
**Health system and information**	18.3 (22.3)		
To be given written information about the important aspects of your care		16/67 (23.9)	
To be given information (written, diagrams, drawings) about aspects of managing your illness and side-effects at home		14/67 (20.9)	
To be given explanations of those tests for which you would like explanations		16/67 (23.9)	
To be adequately informed about the benefits and side-effects of treatments before you choose to have them		11/67 (16.4)	
To be informed about your test results as soon as feasible		16/67 (23.9)	
To be informed about cancer which is under control or diminishing		8/67 (11.9)	
To be informed about things you can do to help yourself get well		21/67 (31.3)	
Access to professional counselling (e.g., psychologist, social worker, counsellor, nurse specialist) if you/family/friends need it		24/67 (35.8)	9/10
To be treated like a person, not just another case		16/67 (23.9)	
To be treated in a hospital or clinic that is as physically pleasant as possible		18/67 (26.9)	
One member of hospital staff with whom you can talk to about all aspects of your condition, treatment and follow-up		23/67 (34.3)	10/10
**Sexuality**	13.4 (19.5)		
Changes in sexual feelings		9/67 (13.4)	
Changes in sexual relationships		7/67 (10.4)	
To be given information about sexual relationships		15/67 (22.4)	

*The summated mean scores were standardised using the formula provided in the SCNS guidelines. The formula was as follows: *a* × 100/(*m* × (*k* − 1)), where *m* is the number of items in a domain; *a* is the adjusted Likert score (crude score − *m*) and *k* is the maximum score value for each item

**Table 4:** Caregiver unmet supportive care needs by domain and individual items of the SCNS-PC

SCNS-P&C items by domain	Standardised mean ( ±)*	Caregivers with unmet need/number of respondents (%)	Top 10 needs
**Psychological and emotional**	28.8 (25.7)		
Managing concerns about recurrence		17/41 (41.5)	= 4/10
The influence cancer has had on your relationship with survivor		6/41 (14.6)	
Understanding the experiences of the survivor		9/41 (22.0)	
Balancing own and survivor’s needs		16/41 (39.0)	= 7/10
Adjustment to changes in survivors body		9/40 (22.5)	
Addressing problems in your sex life		3/40 (7.5)	
Getting emotional support for yourself		16/41 (39.0)	= 7/10
Getting emotional support for the people you love		13/41 (31.7)	
Dealing with your emotions about death and dying		13/41 (31.7)	
Dealing with others who don’t recognise the effects on your life of looking after the survivor		17/41 (41.5)	= 4/10
Dealing with your emotions when the recovery of the person with cancer has not happened as you had expected		12/41 (29.3)	
Making decisions about your life in the midst of uncertainty		17/41 (41.5)	= 4/10
Being able to give meaning to the survivor’s illness		7/41 (17.1)	
Exploring your spiritual beliefs		3/41 (7.3)	
**Information**	27.0 (26.2)		
Information relevant to your carer needs		15/42 (35.7)	
Information about prognosis		6/42 (14.3)	
Information about support services		16/41 (39.0)	= 7/10
Information about alternative therapies		9/41 (22.0)	
Information about survivor physical needs		9/41 (22.0)	
Information about side effects of treatment		12/41 (29.3)	
Information about possible fertility problems		18/42 (42.9)	= 1/10
Information about financial support and governmental benefits		18/42 (42.9)	= 1/10
Information about life and/or travel insurance		15/41 (36.6)	
Information about accessing legal services		8/41 (19.5)	
**Health care service**	26.7 (26.5)		
Getting the best medical care		10/41 (24.4)	
Accessing local health services		13/40 (32.5)	
Being involved in survivor medical care		7/42 (16.7)	
Opportunity to discuss care with doctor		8/42 (19.0)	
Feeling confident that all the doctors consult with each other to coordinate care		12/41 (29.3)	
A case manager who coordinated services		15/42 (38.7)	= 10/10
Complaints regarding care being addressed		6/42 (14.3)	
Reducing stress in the survivor’s life		16/42 (38.1)	
Looking after your own health		16/42 (38.1)	
Pain control for survivor		3/42 (7.1)	
Fears about survivor physical and mental deterioration		15/41 (36.6)	
Managing practical caring tasks		10/42 (23.8)	
Accessing hospital parking		16/42 (38.1)	
The opportunity to participate in decision making about survivors treatment		7/37 (18.9)	
**Work and social**	25.2 (22.6)		
Changes to survivor working life or usual activities		14/42 (36.6)	
Influence of caring on your working life or usual activities		18/42 (42.9)	= 1/10
Communicating with the patient with cancer		9/41 (22.0)	
Communicating with family		6/41 (14.6)	
Getting more support from your family		9/41 (22.0)	
Talking to other cancer carers		6/41 (14.6)	
Discussing the cancer in social situations or at work		9/41 (22.0)	

*The summated mean scores were standardised using the formula provided in the SCNS guidelines. The formula was as follows: *a* × 100/(*m* × (*k* − 1)), where *m* is the number of items in a domain; *a* is the adjusted Likert score (crude score − *m*) and *k* is the maximum score value for each item

Regarding caregivers, the standardised scores (Table 4) indicate that psychological needs had the highest mean score of all the SCNS-P&C domains (28.8 ± 25.7). Also, half of the top 10 ranked unmet needs belonged to the psychological and emotional domain (5/10). The top unmet needs in this domain were ‘*managing concerns about recurrence*’, ‘*dealing with others who don’t recognise the effects on your life of looking after the survivor*’ and ‘*making decisions about your life in the midst of uncertainty*’ (all 41.5%). The information domain was another highly reported area of need (27.0 ± 26.2). Two of the highest ranked items were also in this domain, nearly half of caregivers wanted information about survivor fertility problems and financial support for themselves and/or the survivor (both 42.9%). The lowest standardised mean score was the work and social domain (25.2 ± 22.6). Yet, one of the top ranked unmet needs was in this domain, nearly half (42.9%) of caregivers identified a need for help with ‘*the impact that caring for the survivor has had on their working life, or usual activities*’.

### Association between participant characteristics and supportive care needs

Univariate regression analysis identified six sociodemographic and clinical variables that significantly correlated (*p* < 0.10) with the reporting of survivor unmet needs. Unmet needs were more prevalent in females (*r*^2^ = 0.89, *p* = 0.013), survivors further away from diagnosis (*r*^2^ = 0.28, *p* = 0.090), those not in a relationship (*r*^2^ = 0.42 *p* = 0.092), those not in employment (*r*^2^ = 0.081 *p* = 0.018), those not treated with surgery (*r*^2^ = 0.43 *p* = 0.093) and chemotherapy (*r*^2^ = 0.52 *p* = 0.065). In the final multivariable model, sex, time since diagnosis and employment status remained statistically significantly associated with survivor unmet needs (*r*^2^ = 0.237, *p* < 0.01).

Univariate analysis identified three caregiver variables that significantly correlated (*p* < 0.10) with the reporting of unmet needs (Table 5). The analysis indicated that unmet needs were more prevalent in single caregivers (*r*^2^ = 0.281 *p* < 0.001), caregivers caring for younger survivors (*r*^2^ = 0.079, *p* = 0.079) and those caring for survivors closer to treatment (*r*^2^ = 0.102, *p* = 0.044). In the final multivariable model, only relationship status remained (B =  − 15.556, *r*^2^ = 0.281, *p* < 0.001, CI =  − 23.620, − 7.592). This indicates that single caregivers were more likely to report unmet needs, explaining 28.1% of the variance.

**Table 5:** Associations between overall survivor and caregiver supportive care needs scores and sociodemographic/clinical factors

Model	B	***R***^2^	***p*** value	95% CI
Survivor data				
Univariable analyses				
Sex (male = 0/female = 1)	5.053	0.89	0.013**	1.083 to 9.023
Current age	− 0.032	0.000	0.896	− 0.527 to 0.462
Higher education (0 = no/1 = yes)	2.880	0.028	0.173	− 1.295 to 7.055
In a relationship (0 = no/1 = yes)	− 4.296	0.42	0.092*	− 9.310 to 0.718
In employment (0 = no/1 = yes)	− 4.889	0.081	0.018**	− 8.924 to − 0.855
Time since diagnosis	0.372	0.043	0.090*	− 0.60 to 0.804
Surgery (0 = no/1 = yes)	− 3.938	0.043	0.093*	− 8.545 to 0.670
Chemotherapy (0 = no/1 = yes)	− 3.856	0.052	0.065*	− 7.951 to 0.240
Radiotherapy	0.862	0.002	0.714	− 3.648 to 5.299
Multivariable analyses		0.237		
Sex (male = 0/female = 1)	4.973		0.005**	1.299–8.647
In employment (0 = no/1 = yes)	− 5.704		0.002**	− 9.452 to − 1.955
Time since diagnosis	0.476		0.023**	− 0.086–0.866
Caregiver data				
Univariable analyses				
Caregiver current age	− 0.282	0.021	0.363	− 0.902 to 0.338
Higher education (0 = no/1 = yes)	2.516	0.010	0.541	− 5.744 to 10.775
In a relationship (0 = no/1 = yes)	− 15.556	0.281	0.000**	− 23.620 to − 7.492
In employment (0 = no/1 = yes)	2.342	0.009	0.550	− 5.514 to 10.198
Survivor current age	− 0.718	0.079	0.079*	− 1.525 to 0.088
Time since survivor diagnosis	− 0.781	0.102	0.044**	− 1.540 to − 0.021

*p < 0.10, **p < 0.05

### QoL and supportive care needs

Survivors who had more unmet needs reported a lower QoL (*r* =  − 0.621, *p* < 0.001). All SCNS-SF34 needs domains were also significantly negatively correlated with QoL scores. The correlation coefficients ranged from a moderate negative association between QoL and sexuality needs (*r* =  − 0.358 *p* = 0.003) to a strong negative association between QoL and psychological needs (*r* =  − 0.751, *p* < 0.001).

Caregiver number of unmet needs were also significantly negatively associated with overall QoL score (*r* =  − 0.616, *p* < 001). All the SCNS-P&C needs domains were significantly negatively correlated with QoL. The strongest associations were between QoL and psychological and emotional needs (*r* =  − 0.652, *p* < 0.001). See supplementary information 1 for full QoL analysis data.

## Discussion

This study provides valuable information on the supportive care needs of TYA childhood brain tumour survivors and their caregivers. Over three-quarters (76.8%) of survivors reported at least three unmet needs in long-term survivorship, while over a quarter (27.5%) reported at least 15 unmet needs. The most prevalent unmet needs were in the psychological domain, with around half of all survivors wanting support with anxiety, feeling down or depressed and uncertainty about the future. This highlights that psychological support services should be available not only during treatment but also in long-term survivorship. These findings are consistent with previous studies (mixed cancer cohorts, excluding brain tumours), who proposed a greater unmet need for long-term, post-treatment psychological interventions [28, 29]. There are no other quantitative studies that have looked at unmet needs in TYA survivors of childhood brain tumours [6], so we are unable to compare results directly.

We identified associations between survivor unmet needs and sociodemographic/clinical characteristics. Female survivors were more likely to report more needs. These findings are similar to Boyes and colleagues who looked at unmet needs and survivor characteristics (within mixed cancer survivors) [30]. They found that sociodemographic variables were more significant predictors of unmet needs than clinical ones and that sex (female) was associated with higher supportive care needs in survivors [30]. Additionally, unemployed survivors were more likely to experience unmet needs. There is little in the literature that highlights the association between unemployment and survivor needs. Yet, we know that adult survivors have difficulty securing and maintaining jobs, further indicating the need for support in this area [31]. Survivors further away from treatment were also more likely to experience unmet needs. It is often thought that time since diagnosis mitigates the effects of cancer. Yet, this finding highlights the importance of long-term survivorship care. These findings are similar to Keir et al., who found that long-term adult brain tumour survivors were as likely to be categorised as ‘stressed’ than patients closer to diagnosis [32].

The majority (88.4%) of caregivers experienced at least one unmet need. This number is higher than other studies. Balfe and colleagues found that around half of caregivers caring for an adult brain tumour survivor reported at least one unmet need [33]. Another study found that parents of children in treatment for cancer (mixed diagnoses) reported more unmet needs (83% had over 10 unmet needs) [34] compared to 46.5% in our report. Still, it is striking that the caregivers in this study had so many unmet needs years after treatment. Very few studies have addressed how caregiver problems and needs change throughout the illness trajectory, or how this interacts with changes in social support, QoL, employment and relationships. More longitudinal studies are needed to better understand how caregiver needs vary over time.

Like survivors, caregiver psychological needs were pressing. Yet, two of the most frequent caregiver unmet needs were in the information domain, with nearly half of all caregivers (42.9%) wanting information about survivor fertility, and financial support/governmental benefits. These findings suggest that new information resources should focus on these two areas. These are likely the most frequent needs of this population of caregivers because their loved ones are younger and these are or will soon be pressing issues. These unmet needs differ from caregivers of adult survivors, whose most pressing need was for support managing fears about recurrence [33]. The findings also highlight that long-term supportive services/care should pay attention to caregivers who are not in relationships, as they were more likely to experience unmet needs. This may be due to single caregivers having less informal support and relying more on formal supportive care services.

In both survivors and caregivers, we found moderate/strong negative correlations between unmet needs and poorer QoL. The strongest association was between poor QoL and unmet psychological needs. Again, reiterating psychological care is an essential area to target for significantly improving the general sense of survivors’ and caregivers’ quality of life. Previous studies with other cancer groups have found that addressing unmet needs leads to improved QoL [35, 36].

Our findings suggest that despite NICE guidelines recommending long-term multidisciplinary care and access to specialist care services [37], the psychological aspects of long-term care are not currently being adequately addressed. However, many services are strained and do not have the resources to address psychological concerns throughout long-term survivorship. Other barriers include long waiting lists, issues with referrals and a decline in supportive services as TYAs get older [14]. Therefore, signposting and referrals to psychological services provided in the community (e.g. by brain tumour charities) could be helpful for some survivors and caregivers.

There are few psychosocial intervention studies in the literature targeting long-term childhood cancer survivors, let alone this unique population. A systematic review by Bradford and Chan found a lack of high-quality studies and no conclusive evidence favouring specific interventions [38]. However, they concluded that using technology to deliver interventions is likely to improve delivery. Due to the unique late effects of this population (e.g. cognitive deficits and physical disabilities), we know that the format of information/support should be carefully considered [6]. Our study has also indicated the need for caregiver psychological support. More high‐quality research is needed to develop psychological interventions and test their effectiveness to support family caregivers [39].

This study provides multi-centre, brain tumour specific data, using validated measures. However, there are some limitations. First, sampling bias is a possibility. It may be that some survivors and caregivers did not complete the surveys because they felt unable to (due to high levels of anxiety, depression or cognitive limitations) or because they felt this research did not apply to them (they had zero late effects/needs). Second, most of the sample was recruited from three NHS Trusts located in Yorkshire in the UK, which may limit the generalisations of the findings. Third, the SCNS measures are well-validated tools for investigating multiple dimensions of supportive care needs. However, it is possible that they may not fully capture the unique needs of survivors and caregivers later in the survivorship phase, or those that are brain tumour specific. Therefore, this study may underestimate the prevalence of unmet needs reported by survivors and caregivers.

## Conclusion

There is growing agreement across all cancer types that it is essential to meet the information and supportive care needs of those living with and beyond cancer [40, 41]. The data presented in this article extends the very limited research in this area by gaining an understanding of the supportive care needs of TYA childhood brain tumour survivors and their caregivers. Unmet supportive care needs were common in long-term TYA survivors and their caregivers, with some experiencing a very high number of unmet needs. The unmet needs among both TYA survivors and caregivers were predominantly in the psychological domain. These findings provide comprehensive insight that timely psychological support and interventions should be a high priority to support families in long-term survivorship and ensure a better QoL.

## Supplementary Information

Below is the link to the electronic supplementary material.

Supplementary file1 (DOCX 14 kb)I could not access the link to the supplementary information. 

## Data Availability

Data are held securely by the research team and may be available upon reasonable request and with relevant approvals in place.

## References

[1] Office for National Statistics (2018) Cancer registration statistics, England: 2016- cancer diagnoses and age-standardised incidence rates for all types of cancer by age, sex and region including breast, prostate, lung and colorectal cancer. https://www.ons.gov.uk/peoplepopulationandcommunity/healthandsocialcare/conditionsanddiseases/bulletins/cancerregistrationstatisticsengland/final2016. Accessed 13 January 2021

[2] Ostrom QT, Gittleman H, Truitt G, Boscia A, Kruchko C, Barnholtz-Sloan JS (2018). CBTRUS statistical report: primary brain and other central nervous system tumors diagnosed in the United States in 2011–2015. Neuro Oncol.

[3] Basta NO, James PW, Gomez-Pozo B, Craft AW, McNally RJ (2011). Survival from childhood cancer in northern England, 1968–2005. Br J Cancer.

[4] Anderson DM, Rennie KM, Ziegler RS, Neglia JP, Robison LR, Gurney JG (2001). Medical and neurocognitive late effects among survivors of childhood central nervous system tumors. Cancer.

[5] Turner CD, Rey-Casserly C, Liptak CC, Chordas C (2009). Late effects of therapy for pediatric brain tumor survivors. J Child Neurol.

[6] Nicklin E, Velikova G, Hulme C, Rodriguez Lopez R, Glaser A, Kwok-Williams M, Boele F (2019). Long-term issues and supportive care needs of adolescent and young adult childhood brain tumour survivors and their caregivers: a systematic review. Psychooncology.

[7] Gardner MH, Mrug S, Schwebel DC, Phipps S, Whelan K, Madan-Swain A (2017). Benefit finding and quality of life in caregivers of childhood cancer survivors: the moderating roles of demographic and psychosocial factors. Cancer Nurs.

[8] Witt WP, Litzelman K, Wisk LE, Spear HA, Catrine K, Levin N, Gottlieb CA (2010). Stress-mediated quality of life outcomes in parents of childhood cancer and brain tumor survivors: a case-control study. Qual Life Res.

[9] Bennett E, English MW, Rennoldson M, Starza-Smith A (2013). Predicting parenting stress in caregivers of children with brain tumours. Psychooncology.

[10] Boele FW, Given CW, Given BA, Donovan HS, Schulz R, Weimer JM, Drappatz J, Lieberman FS, Sherwood PR (2017). Family caregivers’ level of mastery predicts survival of patients with glioblastoma: a preliminary report. Cancer.

[11] Wadhwa D, Burman D, Swami N, Rodin G, Lo C, Zimmermann C (2013). Quality of life and mental health in caregivers of outpatients with advanced cancer. Psychooncology.

[12] Grunfeld E, Coyle D, Whelan T, Clinch J, Reyno L, Earle CC, Willan A, Viola R, Coristine M, Janz T, Glossop R (2004). Family caregiver burden: results of a longitudinal study of breast cancer patients and their principal caregivers. CMAJ Can Med Assoc J.

[13] Sanson-Fisher R, Girgis A, Boyes A, Bonevski B, Burton L, Cook P, Group SCR (2000). The unmet supportive care needs of patients with cancer. Cancer.

[14] Nicklin E, Pointon L, Glaser A, Sarwar N, Kwok-Williams M, Debono M, Velikova G, Boele FW (2021). Unmet support needs in teenage and young adult childhood brain tumour survivors and their caregivers: “it’s all the aftermath, and then you’re forgotten about”. Support Care Cancer.

[15] von Elm E, Altman DG, Egger M, Pocock SJ, Gøtzsche PC, Vandenbroucke JP (2007). The Strengthening the Reporting of Observational Studies in Epidemiology (STROBE) statement: guidelines for reporting observational studies. Lancet.

[16] Geiger AM, Castellino SM (2011). Delineating the age ranges used to define adolescents and young adults. J Clin Oncol.

[17] Editors (2011) What should the age range be for AYA oncology? J Adolesc Young Adult Oncol 1: 3–10 10.1089/jayao.2011.1505 26812562

[18] Teenage Cancer Trust. https://www.teenagecancertrust.org/about-us. Accessed 09 Jan 2021

[19] National Cancer Institute. [Internet]. https://www.cancer.gov/types/aya. Accessed 09 Jan 2021

[20] Boyes A, Girgis A, Lecathelinais C (2009). Brief assessment of adult cancer patients’ perceived needs: development and validation of the 34-item Supportive Care Needs Survey (SCNS-SF34). J Eval Clin Pract.

[21] Girgis A, Lambert S, Lecathelinais C (2011). The supportive care needs survey for partners and caregivers of cancer survivors: development and psychometric evaluation. Psychooncology.

[22] McElduff P, Boyes A, Zucca A, Girgis A (2004) Supportive care needs survey: a guide to administration, scoring and analysis. Newcastle: Centre for Health Research & Psycho-Oncology

[23] FACIT. Pediatric functional assessment of cancer therapy – brain tumor survivor (version 2) patient version (age 12 years and older). http://www.facit.org/facitorg/questionnaires. Accessed 23 Mar 2019

[24] Lai J-S, Cella D, Tomita T, Bode RK, Newmark M, Goldman S (2007). Developing a health-related quality of life instrument for childhood brain tumor survivors. Childs Nerv Syst.

[25] Weitzner MA, Jacobsen PB, Wagner H, Friedland J, Cox C (1999). The Caregiver Quality of Life Index-Cancer (CQOLC) scale: development and validation of an instrument to measure quality of life of the family caregiver of patients with cancer. Qual Life Res.

[26] Ratner B (2009). The correlation coefficient: its values range between +1/−1, or do they?. J Target Meas Anal Mark.

[27] Fayers PM, Machin D (2000). Quality of life: assessment, analysis, and interpretation.

[28] Barr RD (2016). Planning a comprehensive program in adolescent and young adult oncology–a collision with reality. J Adolesc Young Adult Oncol.

[29] Fern LA, Taylor RM, Whelan J, Pearce S, Grew T, Brooman K, Starkey C, Millington H, Ashton J, Gibson F (2013). The art of age-appropriate care: reflecting on a conceptual model of the cancer experience for teenagers and young adults. Cancer Nurs.

[30] Boyes AW, Clinton-McHarg T, Waller AE, Steele A, D’Este CA, Sanson-Fisher RW (2015). Prevalence and correlates of the unmet supportive care needs of individuals diagnosed with a haematological malignancy. Acta Oncol.

[31] de Boer AG, Verbeek JH, van Dijk FJ (2006). Adult survivors of childhood cancer and unemployment: a metaanalysis. Cancer.

[32] Keir ST, Swartz JJ, Friedman HS (2007). Stress and long-term survivors of brain cancer. Support Care Cancer.

[33] Balfe M, Maguire R, Hanly P, Butow P, O'Sullivan E, Timmons A, Gooberman-Hill R, Sharp L (2016). Distress in long-term head and neck cancer carers: a qualitative study of carers’ perspectives. J Clin Nurs.

[34] Aziza YDA, Wang S-T, Huang M-C (2019). Unmet supportive care needs and psychological distress among parents of children with cancer in Indonesia. Psychooncology.

[35] Husson O, Mols F, van de Poll-Franse LV (2011). The relation between information provision and health-related quality of life, anxiety and depression among cancer survivors: a systematic review. Ann Oncol.

[36] Cramarossa G, Chow E, Zhang L, Bedard G, Zeng L, Sahgal A, Vassiliou V, Satoh T, Foro P, Ma BB, Chie WC, Chen E, Lam H, Bottomley A (2013). Predictive factors for overall quality of life in patients with advanced cancer. Supportive care in cancer.

[37] NICE (2006) Improving outcomes for people with brain and other CNS tumours, London National Institution of Health and Clinical Excellence. http://www.nice.org.uk/csgbraincns. Accessed 02 Feb 2021

[38] Bradford NK, Chan RJ (2017). Health promotion and psychological interventions for adolescent and young adult cancer survivors: a systematic literature review. Cancer Treat Rev.

[39] Boele FW, Rooney AG, Bulbeck H, Sherwood P (2019). Interventions to help support caregivers of people with a brain or spinal cord tumour. The Cochrane database of systematic reviews.

[40] National Cancer Survivorship Initiative (2020). Living with and beyond cancer: taking action to improve outcomes. https://www.gov.uk/government/publications/living-with-and-beyond-cancer-taking-action-to-improve-outcomes. Accessed 09 Dec 2020

[41] National Cancer Institute (2018). About cancer survivorship research: survivorship definitions. https://cancercontrol.cancer.gov/ocs/statistics/definitions.html. 19 Dec 2020

