# Supplementary material for: Long-term unmet supportive care needs of teenage and young adult (TYA) childhood brain tumour survivors and their caregivers: a cross-sectional survey
Source: Support Care Cancer. 2021 Oct 12;30(3):1981–92. doi: 10.1007/s00520-021-06618-7 (PMC8795012; doi:10.1007/s00520-021-06618-7)
Supplement: Supplementary file 1 — Supplementary file1 (DOCX 14 kb)I could not access the link to the supplementary information. [file 520_2021_6618_MOESM1_ESM.docx]

**Supplimentary Information**

*Table 1 -Quality of life scores in correlation to the total number of unmet needs and individual SCNS-SF34 domains*

| SCNS-SF34 |  | Quality of Life overall score (Peds-FACT-Br (Adolescence)) |
| --- | --- | --- |
| Total number unmet needs | Correlation | -.621*** |
|  | P value | .000 |
|  | N | 68 |
| Sexuality domain | Correlation | -.358** |
|  | P value | .003 |
|  | N | 67 |
| Psychological domain | Correlation | -.751*** |
|  | P value | .000 |
|  | N | 67 |
| Physical and daily living domain | Correlation | -.729*** |
|  | P value | .000 |
|  | N | 68 |
| Patient Care and Support domain | Correlation | -.515*** |
|  | P value | .000 |
|  | N | 67 |
| Health System and Information domain | Correlation | -.449*** |
|  | P value | .000 |
|  | N | 67 |

* p<.05, ** p<.01 ***p<.001

Table 2- Caregiver quality of life score in correlation to total number of unmet needs and individual SCNS-P&C domains

| SCNS-P&C |  | Quality of Life overall score (CQOLC) |
| --- | --- | --- |
| Total number unmet needs | Correlation Coefficient | -.616 |
|  | P value | .000** |
|  | N | 41 |
| Psychological and emotional needs | Correlation Coefficient | -.652 |
|  | P value | .000** |
|  | N | 41 |
| Informational needs | Correlation Coefficient | -.587 |
|  | P value | .000** |
|  | N | 41 |
| Health Care Service needs | Correlation Coefficient | -.602 |
|  | P value | .000** |
|  | N | 41 |
| Work and Social needs | Correlation Coefficient | -.530 |
|  | P value | .000** |
|  | N | 41 |

*** P<.001
